# Characterization of ceftriaxone-resistant *Aeromonas* spp. isolates from stool samples of both children and adults in Southern India

**DOI:** 10.1186/s41043-015-0036-7

**Published:** 2015-12-01

**Authors:** Maanasa Bhaskar, K P Dinoop, Jharna Mandal

**Affiliations:** Department of Microbiology, JIPMER, Puducherry, India

**Keywords:** Aeromonas, Diarrhoea, MIC, ESBL, AmpC beta-lactamase

## Abstract

**Background:**

*Aeromonas* species can cause a wide spectrum of illnesses varying from intestinal to extra intestinal and vary in their susceptibility to different antibiotics. The current study was undertaken to characterize the third generation cephalosporin-resistant strains of *Aeromonas* spp. which were isolated from stool specimens.

**Methods:**

Out of a total of 2780 stool samples, 29 *Aeromonas* spp. were identified, out of which, 9 were resistant to ceftriaxone by the Kirby-Bauer antibiotic testing method. These strains were subjected to minimum inhibitory concentration (MIC) determination by agar dilution for ceftriaxone. Phenotypic and genotypic testing of AmpC beta-lactamase and extended spectrum beta-lactamase (ESBL) were performed. Gene transfer was carried out to demonstrate transmissibility of these genetic elements by conjugation experiments.

**Results:**

Out of the 29 strains, 9 showed MIC of ≥4 μg/ml. Seven out of 9 showed presence of blaCTX-M, while 2 more strains showed the presence of inducible AmpC beta-lactamase and presence of MOX gene. Gene transfer experiments showed that these elements were transmissible to recipient (*Escherichia coli* J53 strain) in the presence of ceftriaxone.

**Conclusions:**

Dissemination of these resistance determinants like plasmids is pivotal in the spread of these resistance genes into the aquatic environment into organisms like *Aeromonas*. This may further limit the future use of antibiotics for the treatment of diarrhoeal diseases. Hence, detection and antibiotic susceptibility testing of *Aeromonas* spp. should be performed when isolated from stool samples.

## Background

The family *Aeromonadaceae* contains the members of the genus *Aeromonas* which are Gram-negative rods, facultative anaerobes and oxidase-positive. They are habitants of a wide range of aquatic environment like fresh, marine and estuarine water, as well as sewage where the bacterial ecosystem co-exists as well as are natural pathogens of aquatic life forms like fishes, etc. [1].

A number of *Aeromonas* species are described, but the taxonomy of the genus *Aeromonas* is confusing because of the lack of congruity between the phenotypic and genotypic characters. Now, several DNA hybridization methods are available based on which *Aeromonas* species are classified [1, 2]. Important *Aeromonas* species which are pathogenic for humans include *Aeromonas hydrophila*, *Aeromonas sobria*, *Aeromonas trota*, and *Aeromonas caviae* [1, 3, 4].

Today, *Aeromonas* species are considered not only pathogenic to cold-blooded animals and fishes, some of the species are pathogenic to animals and humans also, both immunocompetent and immunocompromised. The spectrum of infections caused by *Aeromonas* species varies from various intestinal manifestations to extra intestinal complications namely gastrointestinal tract syndromes, wound and soft tissue infections, urinary tract infections and rarely septicaemia [1, 2].

The role of *Aeromonas* as a gastrointestinal pathogen has been controversial as 1-4 % of asymptomatic individuals are known to harbour them in their gut. However, *Aeromonas*-associated acute bacterial gastroenteritis has been reported from across the world. The gastrointestinal manifestations can vary from an acute gastroenteritis presenting with an acute watery diarrhoea, choleraic illness to a more severe form accompanied by dysentery rarely leading to hemolytic uremic syndrome [1–4].

Susceptibility to the antibiotics varies according to the geographical area and the species of *Aeromonas* tested. Most of the isolates are generally susceptible to tetracyclines and quinolones though development of increasing resistance to third generation cephalosporins due to the production of beta-lactamases and extended spectrum beta-lactamases (ESBLs) has been noted in the recent years [2, 3, 5]. The current study was undertaken to characterize the third generation cephalosporin-resistant strains of *Aeromonas* spp. which were isolated from stool specimens.

## Methods

The present study was a descriptive study conducted in a tertiary care centre in South India. All consecutive non-duplicate stool isolates of *Aeromonas* spp. resistant/intermediate to ceftriaxone by Kirby-Bauer disc diffusion method during a study period of 3 years from January 2010 to December 2012 were included in the study. These isolates were characterized phenotypically and genotypically for determining ceftriaxone resistance mechanisms.

### Bacterial isolates

A total of 2780 stool samples were processed, out of which, 29 (1.04 %) *Aeromonas* spp. were identified. Total samples positive for other pathogens were *Shigella* (6.1 %), non-typhoidal salmonellae (1.29 %) and *Vibrio cholerae* (7 %) among the bacterial causes of diarrhoea, during the study period of 2 years using the cultural characteristics and standard biochemical reactions, followed by confirmation with respective antisera wherever applicable [6, 7]. In these 29 patients, no other pathogen was isolated, and *Aeromonas* spp. were the single pathogen isolated.

### Antimicrobial susceptibility testing

Antimicrobial susceptibility testing of all the isolates was performed on Mueller-Hinton agar plates by standard Kirby-Bauer disc diffusion method as per the standard procedure mentioned in Clinical Laboratory Standards Institute guidelines for *Aeromonas* spp. [8]. The isolates were tested to the following panel of antibiotics including the beta-lactam and the non-beta lactam group of antibiotics namely ceftriaxone (30 μg), ciprofloxacin (5 μg), tetracycline (30 μg), cotrimoxazole (1.25/23.75 μg) and chloramphenicol (30 μg). All the isolates resistant to ceftriaxone were further subjected to minimum inhibitory concentration (MIC) determination for ceftriaxone and characterized phenotypically and genotypically for other cephalosporin resistance mechanisms like AmpCBL (beta-lactamase) production. Since the current updated CLSI guidelines do not necessarily recommend performing ESBL testing routinely with the modified zone diameters for ceftriaxone, we therefore did not perform any further phenotypic tests for the ESBL production.

Determination of MIC—MIC of a particular strain intermediate/resistant to ceftriaxone by routine disc diffusion method was determined using ceftriaxone agar dilution method using ceftriaxone pure powder (Himedia, Mumbai, India). MIC was performed on Mueller-Hinton agar containing doubling dilutions of ceftriaxone ranging from 0.5 to 8 μg/ml. Plates were incubated at 37 °C for 18 h. MIC value of ≤1 μg/ml is considered to be sensitive, 2 μg/ml to be intermediate and ≥4 μg/ml to be resistant to ceftriaxone [6–8].

Detection and identification of various beta-lactamase genes—All the *Aeromonas* species resistant to ceftriaxone by disc diffusion method and further by agar dilution method was further subjected to PCR for identification of various beta-lactamases viz—ESBLs and AmpCBLs.

Preparation of template DNA—DNA was extracted from the isolates by using the boiling method as described previously [9].

ESBL gene detection—Most of the ESBLs genes are variants of blaTEM and blaSHV, but blaCTX-M are also increasingly becoming important. The various genes targeted for detection of ESBLs in the present study were blaTEM,blaSHV and blaCTX-M. A duplex PCR for blaTEM and blaSHV was done using the primer sequence and the cycling conditions of Jemima et.al. [9], and a simple PCR was performed for the detection of blaCTX-M-sing the primer sequence and the cycling conditions as mentioned previously [10]. Amplicons obtained were analyzed by gel electrophoresis using 2 % agarose. Gels were stained by ethidium bromide and visualized by UV transillumination.

Detection of AmpCBL production—The various AmpCBL genes targeted were MOX, CITM, FOX, DHAM, ACCM and EBCM [11]. A multiplex PCR was performed for the detection of these genes using the primer sequence and cycling conditions as described earlier [12]. Sequencing of these genes was conducted by Macrogen Inc. (Seoul, South Korea). We used the BLASTN program (www.ncbi.nlm.nih.gov/BLAST) for database searching.

Transfer of resistance genes—A conjugation experiment for the transferability of the resistance genes was conducted using broth mating experiment with sodium azide resistant *E. coli* strain J53 as the recipient strain as per Lee et al. [13]. Equal volumes (4 ml) of cultures of ceftriaxone-resistant *Aeromonas* strain and *E. coli* strain J53 (each at 109 cfu/ml) grown in brain-heart infusion broth were mixed. Mixtures were incubated at 37 °C for 18 h. Transconjugants were selected on MacConkey agar with sodium azide (150 mg/l) to inhibit the donor strain and with ceftriaxone (1 mg/l) to inhibit the growth of the recipient strain.

Confirmation of transconjugants by PCR amplification—Three to five colonies of the transconjugants were selected to confirm the plasmid transfer of resistance genes. Colonies were picked up from the MacConkey plates containing ceftriaxone (1 mg/l) and DNA extracted from the colonies using boiling lysis method as described above. The supernatant was taken as the template for PCR amplification for detection of AmpCBL and ESBL genes.

## Results

A total of 9 *Aeromonas* species out of 29 strains were found resistant to ceftriaxone by Kirby-Bauer disc diffusion method. All these 9 strains showed MIC of ≥ 4 μg/ml which indicated that they were resistant to ceftriaxone. Seven out of 9 were ESBL producers and showed the presence of blaCTX-M, while 2 more strains showed the presence of inducible AmpCBL. None of these 9 isolates showed the presence of blaTEM or blaSHV. On sequencing these amplicons of blaCTX-M, we found that they had 99 % identity with *E. coli* strain blaCTX-M-15 (Genome accession no: FJ997868.1).

Two of the 9 strains showed the presence of AmpCBL gene at 490 bp position which corresponded to the MOX gene. On sequencing, it was found to match 99 % with *Aeromonas punctate* strain blaMOX-sequence (Genome accession no: FJ262599.1). Co-production of AmpCBL and ESBL was not found in any of our study isolates.

The results of the conjugation experiments followed by the PCR amplification of the transconjugants showed that all 9 strains (7 ESBL producers and 2 AmpCBL producers) were able to transfer their resistance genes to the *E. coli* J53 strain. These tranconjugants were subjected to PCRs for the detection of ESBL and AmpCBL genes, which confirmed the transfer in the 9 strains.

## Discussion

Although the reports of ESBLs associated with *Aeromonas* spp. are rare when compared to the other members of *Enterobacteriaceae* family, the most common type of CTX-M described in India is CTX-M-15. Though there are reports on ceftriaxone resistance in *Aeromonas* from India, molecular characterization studies on ceftriaxone resistance from stool samples are currently not available from India. In our hospital, normally for all watery diarrhoea mainly, rehydration therapy is prescribed, and in case of invasive diarrhoea/dysentery, the antibiotic prescribed usually is cefixime. A lot of ESBLs including TEM-24, TEM-63 and SHV-12 have also been described in *Aeromonas* spp. [14–17].

Previous studies unquestionably established the role (>85 % of isolates) of many *Aeromonas* spp. such as the *A. hydrophila*, *A. caviae* and *A. veronii bv. sobria* in diarrhoea [1, 5].

The *Aeromonas* have been known to carry many drug resistance genes. There are several reports citing the various mechanisms operating. These bacteria can receive and transfer antibiotic resistance genes to other Gram-negative bacteria [2].

The concern for drug resistance in these isolates is well placed as they have originated from the community, causing diarrhoea in the patients discussed. Routinely, antibiotic therapy for *Aeromonas* causing diarrhoea is not warranted, as it produces self-resolving diarrhoea. Hence, the finding of antibacterial resistance is of not a concern as far as diarrhoeal management goes, but since the same pathogen is associated with extra intestinal manifestations where the same resistance mechanisms, operating can spell devastation. Another point of concern is that since these genetic elements are borne on plasmids, they can be transmitted to other bacteria as well which may further limit the future use of antibiotics for the treatment of diarrhoeal diseases due to the latter bacteria. Our plasmid transfer studies have shown that these genetic elements can be easily transmitted from one bacterium to the other each belonging to different genera. This obviously raises concern knowing the fidelity and integrity of such genetic elements. The implications are far extending.

Earlier studies have reflected on the drug resistance mechanisms in this group. In a study from Kolkata, India, it was observed that many of the *Aeromonas* strains were resistant to furazolidone, quinolones and cephalosporins [5]. In fact, multiple drug resistance among *Aeromonas* spp. have been reported from many parts of the world [16, 18]. In both clinical as well as environmental isolates of *Aeromonas*, antibiotic resistance has been observed. In many studies, an increase in resistance was observed among strains of *Aeromonas* isolated from urban effluents being discharged into rivers [17, 19, 20]. It has been demonstrated in earlier studies that household and industrial effluents are known to contain high levels of antibiotics and antibiotic-resistant bacteria of human and animal origin [19–22].

Based on the findings of the PCR and the sequencing, the genes are plasmid-borne which is evident from the plasmid transfer assays. This raises further concern about such elements operating in the environment. *Aeromonas* species producing extended spectrum beta-lactamases are increasingly being reported. Infections caused by *Aeromonas* harbouring ESBL genes and AmpCBL genes is an important problem in developing countries where infections caused by *Aeromonas* is common. Organisms expressing AmpCBL is an important clinical concern since these isolates are usually resistant to all beta-lactam antibiotics except the fourth generation cephalosporins like cefepime, cefpirome and the carbapenems [11]. Detection of this resistance mechanism is important for surveillance, epidemiological studies and hospital infection control because most of these enzymes are plasmid-mediated, and this results in the spread of resistance genes among the members of the same as well as different families. *Aeromonas* spp. in water habitats act as mediators between water and commensal gut bacteria especially members of the *Enterobacteriaceae*, which further act as the progenitors of the most commonly encountered plasmidic-AmpC genes detected in clinical isolates. These genes have been reported from water bodies and drinking water biofilms [23–25] so much so that the presence of genetic elements with sequence homologies to those of bacterial species such as *V. cholerae* and *Photobacterium damselae* have also been found [26].

Like human gut, water sources act as excellent reservoirs where transfer of genetic material including antibiotic resistance genes can occur as demonstrated by earlier workers [11, 24, 26–32]. In strains of *Aeromonas* isolated from the several water bodies demonstrated the presence of AmpCBLs, quinolone resistance determinants and tetracycline efflux gene, which in turn indicate that the water bodies are getting polluted with antibiotics. This might be acting as a selection pressure responsible for selecting out drug-resistant strains in the environment.

The mechanisms of resistance to third generation cephalosporins in *Aeromonas* have been ascribed to the production of beta-lactamases (ESBLs, AmpCBLs and carbapenemases), efflux pumps and alterations in the outer membrane leading to reduced permeability, though in the present study, we did not look for any of the efflux pumps or alterations in the outer membrane.

## Conclusions

Antibiotic usage in the clinical and non-clinical settings is important for the emergence of antibiotic-resistant isolates. ESBL and AmpCBL production are one of the important resistance mechanisms conferring resistance to penicillins and cephalosporins. Emergence of these resistance mechanisms in *Aeromonas* species is important as these organisms are one of the emerging pathogens in both immunocompetent and immunocompromised patients producing a variety of clinical manifestations. Occurrence of ESBLs and AmpCBLs in *Aeromonas* species emphasizes the importance of constant surveillance of clinical isolates for the prevalence of antibiotic resistance genes, though routine testing of stool isolates is not recommended. Dissemination of these resistance determinants due to its location on mobile genetic elements like plasmids plays an important role in the spread of these resistance genes in the environment which may further limit the future use of antibiotics for the treatment of diarrhoeal diseases.
